# Metal-Free Alpha Trifluoromethylselenolation of Carbonyl Derivatives under Batch and Flow Conditions

**DOI:** 10.3390/molecules24040726

**Published:** 2019-02-18

**Authors:** Elisabetta Massolo, Margherita Pirola, Sergio Rossi, Tiziana Benincori

**Affiliations:** 1 Dipartimento di Chimica, Università degli Studi di Milano, Via Golgi 19, 20133 Milano, Italy; elisabetta.massolo@unimi.it (E.M.); margherita.pirola@unimi.it (M.P.); 2 Dipartimento di Scienza e Alta Tecnologia, Università degli Studi Dell’Insubria, Via Valleggio 11, 22100 Como, Italy

**Keywords:** trifluoromethylselenolation, carbonyl compounds, selenium, fluorinated derivatives, flow chemistry

## Abstract

Trifluoromethylselenolated carbonyl compounds represent an emerging class with potential applications in several fields; however, a widespread use of such compound is hampered by the very limited number of strategies for their preparation. In this study we developed a method for the preparation of α-SeCF_3_ substituted carbonyl derivatives using an in situ generated electrophilic ClSeCF_3_ species. We also implemented an in-flow protocol to improve the safety features of the process.

## 1. Introduction

Selenium containing compounds have been shown to be relevant in a number of different areas ranging from health sciences to nanotechnology, through organic synthesis. Selenium has been included in drug design [1], Ebselen being an outstanding example, which is endowed with anti-oxidant, anti-inflammatory, cytoprotective activity, and with potential applications in the treatment of mental disorders [2,3].

**Scheme 1:**
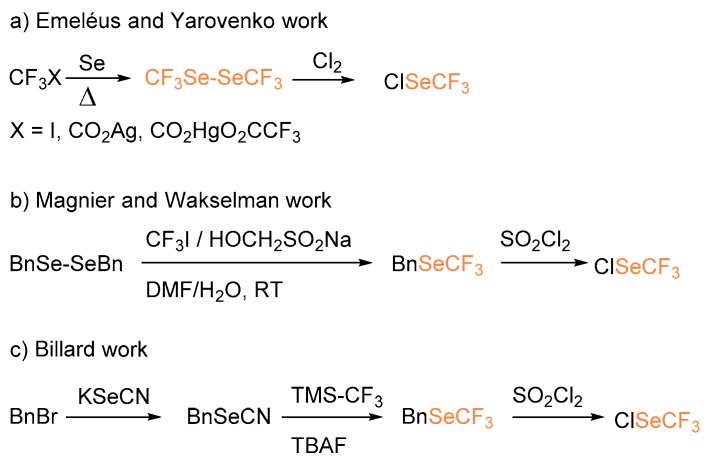
Different approaches for the synthesis of trifluoromethylselenochloride.

Organoselenium derivatives have been appreciated as constituents of self-assembled monolayers [4] substrates used in novel transformations, chiral and achiral organocatalysts, and ligands [5,6,7]. In recent years, new structural units rising from the association between chalcogens and fluorinated moieties have been introduced and studied for their unique properties. Several new reagents have been designed as a source of electrophilic SCF_3_, and a scale of their relative reactivities has been established [7]. However, most of them lack a selenium-containing analogue [8] and, as a consequence, the construction of the C–SeCF_3_ bond has been rarely reported [9]. Similar to the preparation of highly reactive and toxic halogenated trifluoromethylthio derivatives, the in situ generation of trifluoromethylselenochloride species has been developed using different approaches. In particular, in the late 1950s, Emeléus performed the chlorination of bis(trifluoromethyl)diselenide to yield ClSeCF_3_ starting from trifluoroiodomethane and selenium at 260–285 °C [10]. One year later, Yarovenko reported the preparation of hexafluorodimethyl diselenide from trifluoroacetic acid salts (Scheme 1a) [11]. In 2002, Magnier and Wakselman reported the reaction between dibenzyldiselenide and trifluoromethyliodide to afford benzyl (trifluoromethyl)selenide that after treatment with sulfuryl chloride afforded ClSeCF_3_ in 85% overall yield (Scheme 1b) [12]. In a more recent approach, Billard, inspired by the work of Wakselman, developed a convenient strategy for the generation of ClSeCF_3_ starting from benzyl trifluoromethyl selenide (BnSeCF_3_) and SOCl_2_ (Scheme 1c) [13].

ClSeCF_3_ is one of the most widely used reagents used to introduce a SeCF_3_ group into organic molecules, a topic of great interest, as demonstrated by the recent progress toward trifluoromethylselenolation reactions [9,14,15]. Moreover, the high lipophilicity and high electron-withdrawing character of SeCF_3_ group (Hansch lipophilicity parameter σ_R_ = 1.29 compared to π_R_ = 0.88 for CF_3_ group) [16], make it a potential candidate for pharmaceutical and agrochemical applications. As part of our continuing interest in the development of α-chalcogen functionalization of carbonyl compounds [17,18,19] we report herein our study on the introduction of the SeCF_3_ group on enols of various natures, using ClSeCF_3_ as the trifluoromethylselenolating agent. In our approach, we decided to take advantage of the straightforward strategy developed by the Billard’s group for the in preparation of the highly reactive ClSeCF_3_ reagent [20] and studied both the in-batch and the unprecedented in-flow transformation.

## 2. Results and Discussion

**Scheme 2:**
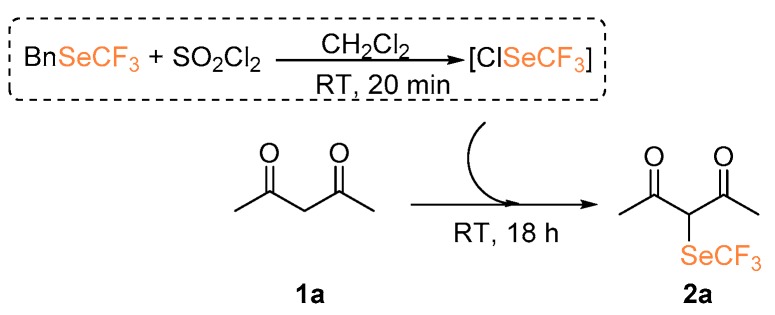
Attempted α-trifluoromethylselenolation of acetoacetone.

At first, we investigated the α-trifluoromethylselenolation of acetoacetone **1a** with the in situ generated ClSeCF_3_ species (Scheme 2). After some preliminary investigations (see Supporting Information), we established the standard reaction conditions to be the following: 18 h at room temperature, dichloromethane as solvent, 1.5 molar equivalents of SO_2_Cl_2,_ and a threefold excess of the chosen nucleophile.

Despite the fact that we observed the formation of compound **2a**, its isolation led to extremely low yields; this could be due to an increased volatility of the reaction product induced by the introduction of the SeCF_3_, in analogy to what has been previously observed in our group in the case of trifluoromethylsulfonylation of ketones [16]. We tested the possibility of functionalizing 3-methyl-acetoacetone **1b**, thus generating a quaternary center (entry 1, Table 1). Also in this case, ^19^F NMR analysis performed on the crude mixture suggested the presence of the properly functionalized diketone **2b**. However, purification and product isolation were unsuccessful. No product isolation was possible even with products derived from 1-pheny-1,3-butandione **1c** and 1,3-indandione **1d** although congruent NMR signals were identified in the crude mixture [21].

**Scheme 3:**
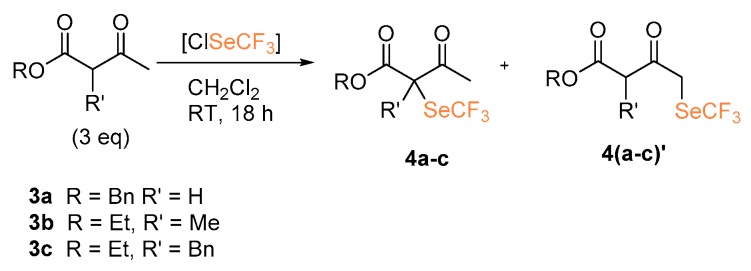
α-Trifluoromethylselenolation of β-keto esters.

The reactions of β-ketoesters and β-diester as nucleophiles were also investigated (Scheme 3). Using benzyl acetoacetate (**3a**) the desired product **4a** was obtained in 16% yield. With the α-substituted ethyl 2-methyl-3-oxobutanoate **3b** and ethyl 2-benzyl-3-oxobutanoate **3c**, functionalization occurred at both C1 and C3 (both products **4b**–**4c** and **4b’**–**4c’** were obtained). Although this does not represent a synthetically useful outcome, it highlights the extreme reactivity of this trifluoromethylselenolating agent: despite the steric hindrance on the more reactive C3 hampering its functionalization, the reaction still proceeds and the less acidic C1 is finally involved in C–Se bond formation.

**Scheme 4:**
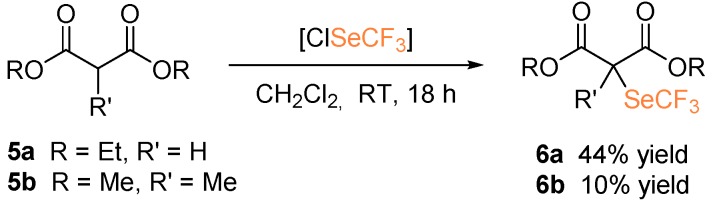
α-Trifluoromethylselenolation of malonates.

Diethylmalonate **5a** and dimethyl methylmalonate **5b** were also subjected to the metal-free trifluoromethylselenolation protocol, in the presence of in situ generated ClSeCF_3_ (Scheme 4). In this case, the desired selenolated product **6a** was isolated in 44% yield. The use of triethylamine as a base to trigger enolate formation was revealed to be detrimental since no formation of the desired product was observed. Functionalization of **5b** for the generation of a quaternary carbon led to the product **6b**, albeit in low yield.

**Scheme 5:**
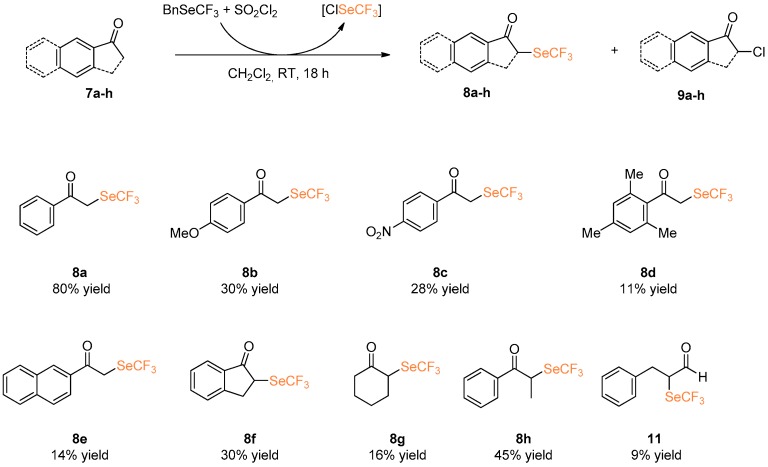
α-Trifluoromethylselenolation of ketones, enamines, silyl enol ethers and aldehydes.

We then focused our attention on the α-trifluoromethlyseleno derivatization of simple ketones **7a**–**f**. Aromatic and aliphatic ketones with different substitutions were investigated in the metal-free α-trifluoroseleno functionalization by reaction with ClSeCF_3_ species (Scheme 5).

Under these conditions, trifluoromethylselenated compound **8a**, derived from propiophenone **7a** was isolated in 80% yield. However, the presence of either an electron donating or withdrawing group on the aromatic ring caused a decrement in terms of yield, as demonstrated by the lower yields obtained using compounds **7b** and **7c**. Steric hindrance plays a fundamental role in the α-trifluoroseleno functionalization: under standard experimental conditions, the reaction of 2,4,6-trimethylacetophenone **7d** leads to the formation of desired **8d** in 11% yield only. Aromatic cyclic ketones such as 2-acetonaphthone **7e** and 1-indanone **7f** afforded the corresponding α-trifluoromethylselenolated compounds **8e** and **8f** in 14% and 30% yield, respectively. In all these cases, purification via column chromatography enabled isolation of the desired product, and in addition chlorinated by-products **9** and unreacted starting material [22].

The use of less reactive aliphatic ketones such as cyclohexanone **7g** led to the formation of the corresponding product **8g** in a yield lower than 16%. The trifluoromethylselenolated derivative of propiophenone (**8h**) was isolated in 45% yield. Attempts to functionalize aldehyde substrates such as hydrocinnamic aldehyde **10** were also performed, but the desired product **11** was isolated in less than 9% yield.

The modest to low yields were most likely due to suboptimal experimental conditions. Indeed, the reactions were performed on a very small scale, as proof of concept of the possibility to realize such transformations, while full information about the toxicity of these selenium-containing compounds is not available yet.

**Figure 1:**
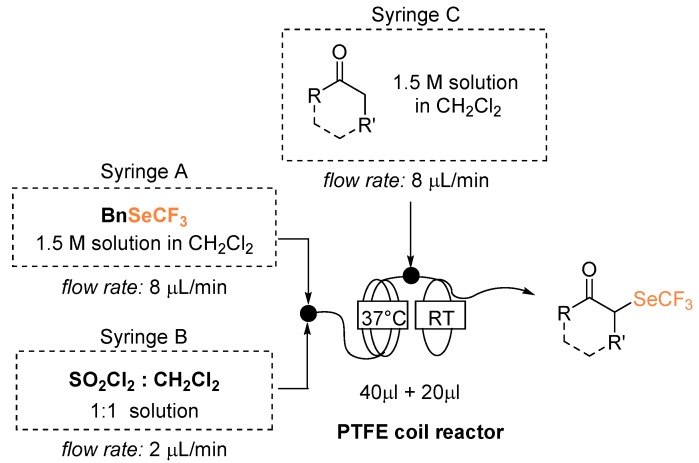
α-Trifluoromethylselenolation under flow conditions. *Reaction temperature* 1st step: 37 °C, 2nd step: RT. *Flow rate* inlet A and C: 8 μL/min, inlet B: 2 μL/min; total flow rate: 18 μL/min. *Residence time* 1st reactor 4 min; 2nd reactor 1.1 min; total residence time 5.1 min.

We reasoned that, to improve the safety aspects, the implementation of an in-flow version would have been a valuable alternative. Our aim was thus to set the basis for scale up under continuous conditions, generating and trapping the low boiling ClSeCF_3_ species in a confined environment. A modular system was conceived where, at the end of a first section of hosting the reaction between BnSeCF_3_ and SO_2_Cl_2_ (PTFE coil, 40 μL volume with internal diameter of 0.07874 cm) the nucleophile’s inlet would be inserted and the α-derivatization would occur in a second reactor section (PTFE coil, 20 μL volume with internal diameter of 0.07874 cm) (Figure 1). In building the flow reactor, material compatibility needs to be evaluated first; as chlorine is evolved, polytetrafluoroethylene (PTFE) is required [23]. With the available equipment, we realized a mesoreactor small enough to avoid the handling, accumulation, and production of selenolated substances. Considering such a small reaction volume (60 μL total volume), low flow rates were needed to allow an adequate residence time.

On the basis of preliminary observations, the effectiveness of the generation of the ClSeCF_3_ could be achieved by running the first step at a temperature slightly higher than RT. Flow rate and reactants concentrations were progressively optimized. In particular, we observed that SO_2_Cl_2_ could not be added neat due to its high density hampering effective addition under our conditions of low flow rates. Besides, a tetrahydrofuran (THF) solution revealed unsuitable, as this solvent probably promotes its deactivation before entering the reaction mixture.

With propiophenone as a model nucleophile, conversion to the desired product improved from 70% to >99%, when raising the temperature of the first step from 37 to 41 °C. An increase in the temperature, however, causes a higher gas generation and induces irregular discontinuities in the reaction flow, thus negatively affecting the process reproducibility. To limit the occurrence of this hard to control microfluidics alteration without excessively hindering the redox step, we set 37 °C as an acceptable compromise. The applied addition rate was the smallest possible to allow a sufficient residence time without altering flow regularity. Optimized reaction conditions are shown in Figure 1; stationary conditions were achieved after a minimum of four reaction volumes.

With these optimized parameters, trifluoromethyl selenolated derivatives were obtained from acetophenone and cyclohexanone in 70% and 80% NMR yield, respectively (Table 2, ketones added neat). When using *p*-methoxy- and *p*-nitroacetophenone (**7b** and **7c**), side reactions occurred leading to fluorinated by-products [24]. We rationalized this result by considering slower C–Se bond formation, possibly due to the solid ketones being added as solutions [25]. We reasoned the formation of ClSeCF_3_ was not completed in the first reactor and was pushed forward by its trapping by the nucleophile; therefore, when an inefficient enolate attack occurs, unreacted BnSeCF_3_, ClSeCF_3_, SO_2_Cl_2_ and its decomposition products coexist for a longer reaction time and undertake undesired pathways. This trend was confirmed when using the poorly nucleophilic diethylmalonate. With β-ketoester **3a**, instead, clean 44% conversion to the selenolated product was observed.

## 3. Materials and Methods

^1^H Reactions were monitored by analytical thin-layer chromatography (TLC) using silica gel 60 F_254_ pre-coated glass plates (0.25 mm thickness) and visualized using UV light. Flash chromatography was carried out on silica gel (230–400 mesh). Proton NMR spectra were recorded on spectrometers operating at 300 MHz (Bruker Fourier 300, Billerica, MA, USA); proton chemical shifts are reported in ppm (δ) with the solvent reference relative to tetramethylsilane (TMS) employed as the internal standard (CDCl_3_, δ = 7.26 ppm). ^13^C-NMR spectra were recorded on 300 MHz spectrometers (Bruker Fourier 300) operating at 75 MHz, with complete proton decoupling; carbon chemical shifts are reported in ppm (δ) relative to TMS with the respective solvent resonance as the internal standard (CDCl_3_, δ = 77.0 ppm). ^19^F-NMR spectra were recorded on 300 MHz spectrometers (Bruker Fourier 300) operating at 282.1 MHz; fluorine chemical shifts are reported in ppm (δ) relative to CF_3_Cl with the respective solvent resonance as the internal standard (CDCl_3_, δ = 77.0 ppm). Mass spectra and accurate mass analyses were carried out on a VG AUTOSPEC-M246 spectrometer (double-focusing magnetic sector instrument with EBE geometry) equipped with EI source or with LCQ Fleet ion trap mass spectrometer, ESI source, with acquisition in positive ionization mode in the mass range of 50–2000 *m*/*z*. The high volatility of the products did not always allow the residence of the sample in the source for a long enough time to record high-res spectra; in these cases, the mass of the products was nevertheless checked by low-resolution mass experiment. The fluidic device was realized by assembling one coil-reactor; reactors were connected, according to Figure 1, by T-junctions using standard HPLC connectors. Coil-reactors consisted of PTFE tubing (diameter: 0.07874 cm) coiled in a bundle. Syringe pump: Chemix Fusion 100, equipped with two 1 mL Hamilton gastight syringes.

### 3.1. General Procedure for the Metal-Free α-Trifluoromethylselenolation of Carbonyl Derivatives under Batch Conditions

Benzyltrifluoromethyl selenide (0.1 mmol, 1.0 equivalents) was dissolved in dry CH_2_Cl_2_ (100 µL, 1 M) and SO_2_Cl_2_ (14 µL, 1.5 equivalents) was added. The reaction mixture was stirred at RT for 20 min, then the nucleophile (3 equivalents) was added. The resulting mixture was stirred for 18 h. The reaction was then partitioned between a saturated water solution of NH_4_Cl and CH_2_Cl_2,_ and the aqueous phase was extracted with CH_2_Cl_2_. The combined organic phases were dried over Na_2_SO_4_. The solvent was removed under moderate vacuum and the crude was analyzed via ^1^H-NMR and ^19^F-NMR. The crude was purified by flash column chromatography on silica gel eluting with the proper pentane/Et_2_O mixture. The desired product was isolated and the solvent removed by flushing air.

### 3.2. General Procedure for the Metal-Free α-Trifluoromethylselenolation of Carbonyl Derivatives under Flow Conditions

The flow reactor was fed by a continuous flow of reagents and reactants: Hamilton gastight syringes were loaded with the needed starting material, whose addition at the defined constant rate was guaranteed by syringe pumps. A 1.5 M solution of (trifluoromethyl)selanylbenzene in CHCl_3_ was loaded in syringe A; a 6.2 M solution of SO_2_Cl_2_ in CHCl_3_ was loaded in syringe B; the ketone (neat when liquid, as a solution in CHCl_3_ when solid) was loaded in syringe C. The first flow reactor (PTFE tube, 40 μL volume) was kept at 37 °C in a thermostated water bath; the second flow reactor (PTFE tube, 20 μL volume) was kept at room temperature. The reaction mixture flow was collected on a silica pad placed at the exit of the reactor.

## 4. Conclusions

In conclusion, the α-trifluoromethlyseleno functionalization of carbonyl derivatives was studied, using the in situ generated highly reactive species ClSeCF_3_. A safe and simple experimental procedure was applied for the straightforward introduction of a SeCF_3_ unit in α-position to a carbonyl group. Investigating different carbonyl derivatives, modest to good yields were obtained. For the first time, a continuous process implemented in a tailor-made modular flow reactor allowed the reaction to be run under controlled conditions, improving the safety of the process. Despite further studies needing to be undertaken to improve the synthetic utility and to widen the reaction scope, this approach already allows the preparation of highly versatile building blocks, useful as starting material for further synthetic elaborations, with potential for application in several fields, as well as opening the pathway to the discovery of novel products of pharmaceutical interest.

## Figures and Tables

**Table 1:** α-Trifluoromethylselenolation of diketones.

Entry	Reagent	Desired Product
1	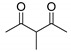 **1b**	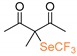 **2b**
2	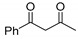 **1c**	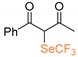 **2c**
3	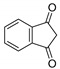 **1d**	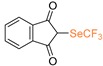 **2d**

**Table 2:** α-Trifluoromethylselenolation of ketones performed under flow conditions.

Entry	Substrate	^19^F-NMR Conversion (%) ^1^
1	**3a**	44
2	**7a**	70
3 ^2^	**7b**	41
4 ^2^	**7c**	23
5	**7g**	80
6 ^3^	**7h**	70

^1^ Calculated using PhCOCF_3_ as internal standard. ^2^ [ketone] = 2 M in dry CH_2_Cl_2_. ^3^ [BnSeCF_3_] = 1 M in dry CHCl_3_.

## Supplementary Materials

Characterization and purification of compounds synthesized in the text is reported in details in the Electronic Supplementary Material.
